# No dose adjustment required for warfarin or metformin when coadministered with the novel GLP-1 receptor agonist ecnoglutide: an open-label, fixed-sequence, crossover study

**DOI:** 10.3389/fphar.2026.1816593

**Published:** 2026-04-29

**Authors:** Zhuo Chen, Chunfeng Du, Linrui Cai, Shiyin Feng, Qin Zou, Weiyi Guo, Fengshan Li, Dan Du, Feng Hu, Xiaohong Liu, Yalin Huang, Shuxia Shi, Wei Shi, Qin Yu, Lingli Zhang, Lei Guan, Liu Yang, Yao Li, Rui Liu, Jing Ning, Ming Yang, Qing Zheng

**Affiliations:** 1 Institution of National Drug Clinical Trial, West China Second University Hospital, Sichuan University, Chengdu, China; 2 NMPA Key Laboratory for Technical Research on Drug Products in vitro and in vivo Correlation, Chengdu, China; 3 Children’s Medicine Key Laboratory of Sichuan Province, Chengdu, China; 4 Key Laboratory of Birth Defects and Related Diseases of Women and Children, Sichuan University, Ministry of Education, Chengdu, China; 5 Evidence-Based Pharmacy Center, West China Second University Hospital, Sichuan University, Chengdu, China; 6 Hangzhou Sciwind Biosciences Co., Ltd., Hangzhou, Zhejiang, China

**Keywords:** drug-drug interaction, ecnoglutide (XW003), GLP-1 receptor agonist, metformin, warfarin

## Abstract

**Objective:**

To characterize the drug-drug interaction (DDI) profile of the novel, long-acting GLP-1 receptor agonist ecnoglutide (XW003) with two widely co-prescribed drugs: metformin and narrow therapeutic index agent, warfarin.

**Methods:**

In this open-label, fixed-sequence, crossover study, 28 healthy participants received a single dose of warfarin and multiple doses of metformin, both with and without steady-state XW003 coadministration. The primary endpoint was the comparison of systemic exposure, assessed by whether the 90% confidence intervals (CIs) for the geometric mean ratios (GMRs) of key pharmacokinetic (PK) parameters fell within the 80.00%–125.00% no-effect bounds. The pharmacodynamic (PD) impact on warfarin was evaluated via International Normalized Ratio (INR).

**Results:**

Coadministration of XW003 did not clinically alter the total systemic exposure (AUC) of metformin or of either S- or R-warfarin enantiomer, as all corresponding GMRs and 90% CIs for AUC met the pre-specified no-effect criteria. While modest reductions in C_max_ and delays in T_max_ were observed for both object drugs, warfarin’s anticoagulant activity remained unaffected, with INR profiles showing no clinically relevant changes. The safety profile of XW003 was consistent with the GLP-1 receptor agonist class.

**Conclusion:**

The findings demonstrate the absence of a clinically significant interaction between XW003 and either metformin or warfarin. This supports the concomitant use of XW003 with these medications without dose adjustment, providing crucial evidence for its safe application in real-world clinical practice where polypharmacy is common.

## 1 Introduction

Glucagon-like peptide-1 (GLP-1) is an incretin hormone secreted by intestinal L-cells postprandially. It stimulates glucose-dependent insulin secretion and suppresses glucagon release. Additionally, it delays gastric emptying and inhibits appetite (Bush et al., 2012). Owing to the widespread distribution of GLP-1 receptors, GLP-1 receptor agonists (GLP-1 RAs) exert broad pharmacological effects, including reduced food intake and body weight (Gabery et al., 2020), cardiovascular protection (Urquhart and Willis, 2020), renoprotection (Clark, 2024), enhanced cognition and learning (Monney et al., 2023), modulation of lipid metabolism (Yao et al., 2024), and regulation of serum uric acid (Najafi et al., 2022). Endogenous GLP-1 is rapidly degraded by dipeptidyl peptidase-4 (DPP-4) and cleared renally, resulting in a very short plasma half-life of approximately 1–2 min (Müller et al., 2019).

XW003 is a novel, long-acting GLP-1 receptor agonist developed through structural modification of the native GLP-1 peptide. This includes amino acid substitution at the DPP-4 cleavage site and conjugation with a modified long-chain fatty acid (Guo et al., 2023). These modifications confer resistance to DPP-4-mediated degradation and facilitate albumin binding, supporting a once-weekly dosing regimen. In clinical studies, XW003 has been administered at doses ranging from 0.3 mg to 1.2 mg once weekly, with 1.2 mg being the therapeutic maintenance dose for type 2 diabetes and weight management. Accordingly, the 1.2 mg steady-state dose was selected for this DDI evaluation. As GLP-1 RAs delay gastric emptying and XW003 has an extended half-life of approximately 124–138 h, coadministration may alter the absorption profiles of concomitant oral medications. Therefore, drug-drug interaction (DDI) studies using representative oral drugs commonly prescribed in the target patient populations are warranted. XW003 is being developed for type 2 diabetes mellitus (T2DM) (Zhu et al., 2024; He et al., 2025), weight management (Ji et al., 2025), and nonalcoholic steatohepatitis (NASH). To inform its use in these contexts, two widely co-prescribed medications were selected as substrate drugs: metformin and warfarin. Metformin, a first-line therapy for T2DM, represents a Biopharmaceutics Classification System (BCS) Class III drug primarily eliminated via renal secretion. Warfarin, a narrow therapeutic index (NTI) anticoagulant (Kimmel, 2008), is metabolized by CYP450 enzymes and serves as a sensitive probe for assessing potential PK interactions that could have serious clinical consequences. Thus, this study was designed to evaluate the DDI potential of XW003 with these two therapeutically distinct and clinically important medications.

## 2 Materials and methods

### 2.1 Ethics approval

This study was registered at ClinicalTrials.gov (NCT06335134) and conducted at the Phase I Clinical Trials Center of West China Second University Hospital (Chengdu, China). The study was performed in compliance with the Declaration of Helsinki and Good Clinical Practice (GCP) guidelines. The protocol and informed consent form were approved by the Clinical Trial Ethics Committee of West China Second University Hospital (Approval Number: Y2023022). Written informed consent was obtained from all participants prior to the initiation of any study procedures.

### 2.2 Study drugs

The investigational medicinal products (IMPs) were provided by Sciwind Biosciences Ltd. (Hangzhou, China). These included Metformin Hydrochloride Immediate-Release Tablets (Glucophage®), Warfarin Sodium Tablets (Cipwar®), and XW003 injection. XW003 was administered via subcutaneous injection in the abdomen, while the other IMPs were administered orally with 240 mL of water.

### 2.3 Study design

**FIGURE 1:**
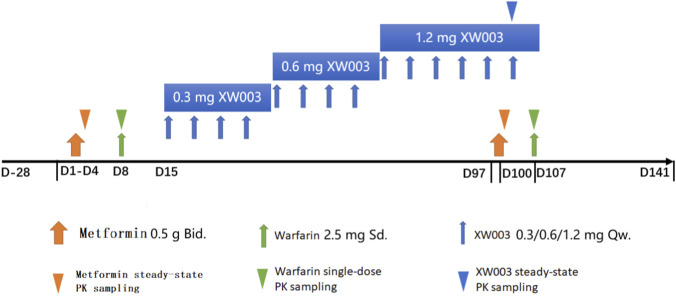
Trial flow diagram.

This open-label, fixed-sequence, crossover study investigated the effects of multiple subcutaneous doses of XW003 (1.2 mg at steady state) on the PK of multiple-dose metformin and single-dose warfarin, as well as the safety, tolerability, and PD of warfarin during concomitant administration (trial procedures and study design are shown in Table 1; Figure 1, respectively). XW003 was initiated at 0.3 mg once weekly, with the dose escalated every 4 weeks to 0.6 mg and then to 1.2 mg, followed by an additional 2 weeks of continued dosing to ensure that drug-drug interactions were assessed under XW003 steady-state conditions. Thus, participants received 4 weeks of 1.2 mg XW003 treatment prior to the metformin DDI assessment and 5 weeks of 1.2 mg treatment prior to the warfarin DDI assessment.

**TABLE 1:** Study procedures.

Timepoint (Day)	D-28 ∼ D-2	D-1	D1	D2	D3	D4	D5	D7	D8	D9	D10	D11	D12	D13	D14	D15
Body Weight Measurement^a^	**×**		**×**						**×**							**×**
Administration																
Metformin^b^			**×**	**×**	**×**	**×**										
Warfarin^c^									**×**							
XW003^d^																**×**
Blood Draw																
Metformin^e^						**×**	**×**									
Warfarin^f^									**×**	**×**	**×**	**×**	**×**	**×**	**×**	**×**
XW003^g^																**×**
INR^h^									**×**	**×**	**×**	**×**	**×**	**×**	**×**	**×**
ADA/Nab																**×**

D22 ∼ D92	D96	D97	D98	D99	D100	D01	D105	D106	D107	D108	D109	D110	D111	D112	D113	D114	D141
**×**		**×**						**×**								**×**	**×**
																	
		**×**	**×**	**×**	**×**												
									**×**								
**×**				**×**				**×**									
																	
					**×**	**×**											
									**×**	**×**	**×**	**×**	**×**	**×**	**×**	**×**	
**×**				**×**	**×**	**×**		**×**	**×**	**×**	**×**	**×**	**×**	**×**	**×**		**×**
									**×**	**×**	**×**	**×**	**×**	**×**	**×**	**×**	
																	**×**

^a^ Body weight was measured under fasting conditions at the following timepoints: Screening, Day 1, Day 8, Day 15, Day 43, Day 71, Day 97, Day 106, Day 114, and at Study Completion/Early Discontinuation.

^b^ Metformin (0.5 g/tablet) was administered at a dose of one tablet (0.5 g) twice daily (morning and evening) for a total of 7 doses per treatment period. The first 6 doses were administered within 1 hour after breakfast (08:00 ± 1:00 h) and dinner (20:00 ± 1:00 h), respectively. The 7th dose was administered in the morning under fasting conditions.

^c^ Warfarin Sodium (2.5 mg/tablet) was administered as a single dose of one tablet (2.5 mg) in the morning under fasting conditions.

^d^ XW003 injection was administered subcutaneously once weekly. The dosing regimen initiated at 0.3 mg for 4 weeks, was then titrated to 0.6 mg for another 4 weeks, and further titrated to the target maintenance dose of 1.2 mg for 6 weeks. The total treatment duration was 14 weeks.

^e^ PK blood samples for metformin were collected from pre-dose until 30 h after the last metformin administration.

^f^ PK blood samples for warfarin sodium were collected from pre-dose until 168 hours after the single warfarin dose administration.

^g^ PK blood samples for XW003 injection were collected according to the protocol.

^h^ Blood samples for the PD analysis of warfarin sodium (International Normalized Ratio, INR) were collected.

Twenty-eight participants received oral metformin (0.5 g) twice daily from Day 1 to the morning of Day 4 (seven doses total). PK blood samples were collected before and after the seventh dose of metformin hydrochloride. On Day 8, participants received a single oral dose of warfarin sodium tablets (2.5 mg), and PK blood samples were collected before and after administration.

During the XW003 coadministration phase, metformin hydrochloride tablets (0.5 g) were administered starting 48 h before the fifth 1.2 mg XW003 dose, twice daily for 3.5 days (seven doses total). PK blood samples were collected before and after the seventh metformin dose. Twenty-four hours after the sixth 1.2 mg XW003 dose, participants received a single oral dose of warfarin sodium tablets (2.5 mg), and PK blood samples were collected before and after administration.

Participants fasted for at least 10 h before warfarin and the last metformin administration. Water was restricted for 1 h before and 2 h after drug administration, and food was restricted for 4 h after administration. The first six metformin doses were administered within 1 h after finishing breakfast (08:00 ± 1:00) and dinner (20:00 ± 1:00), respectively.

### 2.4 Participants

Participants were screened between Days −28 and −2 and subsequently admitted to the research unit on Day −1. Eligible participants included males and non-pregnant, non-lactating females, aged 18–45 years, who weighed ≥50 kg and had a body mass index (BMI) of 20.0–30.0 kg/m^2^. This BMI range (20.0–30.0 kg/m^2^) was chosen to reflect the intended treatment population for XW003, which includes individuals with normal weight, overweight, or obesity, as the drug is being developed for type 2 diabetes and weight management. All participants were required to use highly effective contraception from screening until 3 months after treatment completion. Exclusion criteria comprised any medical condition, clinical or laboratory abnormality, or lifestyle factor that could increase participant risk or interfere with the interpretation of study results, as well as participation in another clinical trial within 3 months prior to the study.

### 2.5 Blood sampling and analysis

For the steady-state PK analysis of metformin, blood samples were collected at 19 time points, ranging from 1 h before to 30 h after the seventh dose. For the single-dose PK analysis of warfarin, samples were taken at 24 time points, from 1 h before to 168 h after administration. PD blood samples for INR measurement (PD assessment) were collected at the same time points as the PK samples. For the PK analysis of XW003, blood samples were collected at 16 time points: 1 h before the first, fifth, ninth, 12th, and 13th injections; 24 and 48 h after the 13th injection; and from 1 h before to 840 h after the 14th injection. Samples for anti-drug antibody (ADA) analysis against XW003 were collected at two time points only: 1 h before the first injection and 840 h after the 14th injection.

At each sampling time point, 3 mL of blood was collected for PK analysis and 2 mL for INR measurement. Blood samples were centrifuged at 1700 g for 10 min at 4 °C within 60 min of collection; plasma was separated and stored at −60 °C until analysis.

The complete blood sampling schedules for metformin, warfarin, and XW003 have been provided as Supplementary Tables S1–S3, respectively, and the full bioanalytical methods (including instrumentation, sample processing, HPLC conditions, MS conditions, and calibration models) have been provided in Supplementary File S1 (Bioanalytical Methods).

A participant was considered evaluable for PK analysis if they had received all required study drug administrations, completed the safety and PK assessments per protocol, and had no major protocol deviations. Plasma PK parameters were estimated from concentration-time profiles using non-compartmental analysis (Phoenix WinNonlin version 8.3; Certara USA Inc.). AUC was calculated using the linear trapezoidal rule. For single-dose administration, the assessed parameters included: maximum observed concentration (C_max_), time to C_max_ (T_max_), area under the concentration-time curve from time zero to the last measurable concentration (AUC_0-last_), AUC from time zero extrapolated to infinity (AUC_0-inf_), percentage of AUC extrapolated from the time of the last measurable concentration to infinity (%AUC_extrap_), apparent terminal half-life (t½), apparent total clearance (CL/F), apparent volume of distribution (Vz/F), and renal clearance (CLr). For multiple-dose administration at steady state, the evaluated parameters included the steady-state analogues of the aforementioned parameters (e.g., C_max,ss_, AUC_0-inf,ss_, AUC_0-last,ss_, t½,ss, CL/Fss, Vz/Fss), as well as the minimum concentration at steady state (Cmin,ss), the average plasma concentration over the dosing interval at steady state (Cavg,ss), and the degree of fluctuation.

The PD analysis was based on INR measurements obtained at each sampling time point before and after warfarin administration. The area under the effect curve (AUC_INR,0-t_), maximum observed INR (INR_max_), and time to reach INR_max_ (T_INRmax_) were derived as observed values. Natural log-transformed data for iAUC_INR,0-t_ and INR_max_ were analyzed using a mixed-effects model. GMRs with 90% confidence intervals (CIs) were calculated for the XW003 coadministration group versus the warfarin-alone group. The 90% CIs of the GMRs for AUC_INR,0-t_ and INR_max_ were evaluated against the standard bioequivalence acceptance range (80.00%–125.00%) to assess the clinical relevance of any interaction.

### 2.6 Safety and tolerability assessments

The safety population comprised all participants who received at least one dose of any study drug. Safety was evaluated through continuous monitoring of adverse events (AEs), 12-lead electrocardiograms (ECGs), physical examinations, vital signs, and clinical laboratory tests throughout the trial.

All serum samples were collected at protocol-specified time points, both pre-dose and post-dose, and were used to detect anti-drug antibodies (ADAs) against XW003. The proportion of participants who developed ADA positivity will be summarized. If applicable, further *in vitro* characterization of ADA-positive samples will be performed.

### 2.7 Statistical analysis

The sample size calculation was based on a two one-sided test procedure with α = 0.05 and 90% power (β = 0.1). Key assumptions included an intra-subject coefficient of variation (CV) ≤20% for metformin steady-state AUC and ≤10% for warfarin AUC. Assuming a geometric mean ratio (GMR) for AUC_0–inf_ between 0.95 and 1.05 with 90% confidence intervals falling within the 80.00%–125.00% bioequivalence range,a minimum of 20 participants was required to complete the study. To accommodate an anticipated 30% dropout rate during this extended study, the final sample size was increased to 28 subjects.

The analysis datasets included the safety set (SS), full analysis set (FAS), pharmacokinetic concentration set (PKCS), pharmacokinetic parameter set (PKPS), and pharmacodynamic parameter set (PDPS) (see Supplementary Table S4 for definitions). The statistical analysis plan encompassed the evaluation of participant disposition, demographic data, baseline characteristics, drug exposure, treatment compliance, PK, PD, immunogenicity, and safety profiles. All analyses were performed using SAS (version 9.4 or higher).

## 3 Results

### 3.1 Demographics and disposition

The study enrolled and completed 28 participants (Supplementary Figure S1). For the pharmacokinetic analyses, 28 participants contributed evaluable data for warfarin and XW003. For metformin, six participants were excluded from the primary PK analysis due to emesis within twice the median T_max_ after the last metformin dose during the XW003 phase; therefore, 22 participants provided evaluable metformin PK data. Table 2 summarizes the demographic and baseline characteristics of the study population.

**TABLE 2:** Demographic characteristics.

Characteristics	Values (N = 28)
Mean age, years SD [Range]	28.7 5.88 [21,44]
Sex, n (%) Male Female	18 (64.29) 10 (35.71)
Race, n (%) Asian	28 (100)
Mean height, cm SD [Range]	165.57 8.65 [149.0, 179.9]
Mean weight, kg SD [Range]	74.15 7.78 [62.1, 91.4]
Mean BMI, kg/m^2^ SD [Range]	27.34 1.16 [25.5, 29.6]
Mean INR SD [Range]	1.04 0.05 [0.94, 1.15]
Glycated Hemoglobin, % SD [Range]	4.95 0.28 [4.27, 5.39]

Abbreviations: SD, standard deviation; BMI, body mass index.

### 3.2 PK

#### 3.2.1 Metformin PK

**FIGURE 2:**
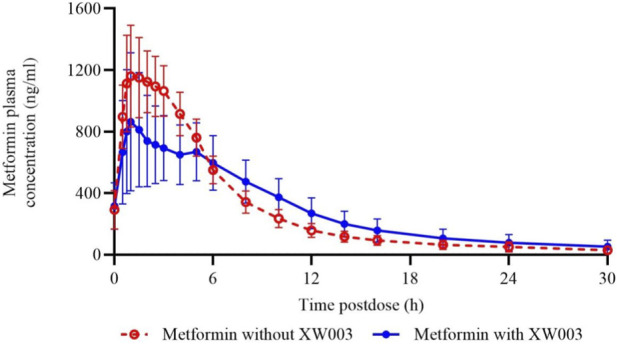
Arithmetic mean plasma concentration-time profiles of metformin at stable-state with or without XW003. Error bars represent standard deviation (SD).

Within the PKCS, blood samples for metformin analysis were collected over 30 h following the last dose, administered with or without XW003 coadministration. Plasma metformin concentrations were quantified using a validated LC-MS/MS method (LLOQ: 3.00 ng/mL). Predose (0 h) concentrations were comparable between treatments. From 0.5 to 5 h post-dose, metformin concentrations were lower with XW003 coadministration than with metformin alone, whereas beyond 5 h, concentrations with XW003 exceeded those without. By 30 h, concentrations returned to comparable levels between the two cohorts (Figure 2).

#### 3.2.2 Warfarin PK

**FIGURE 3:**
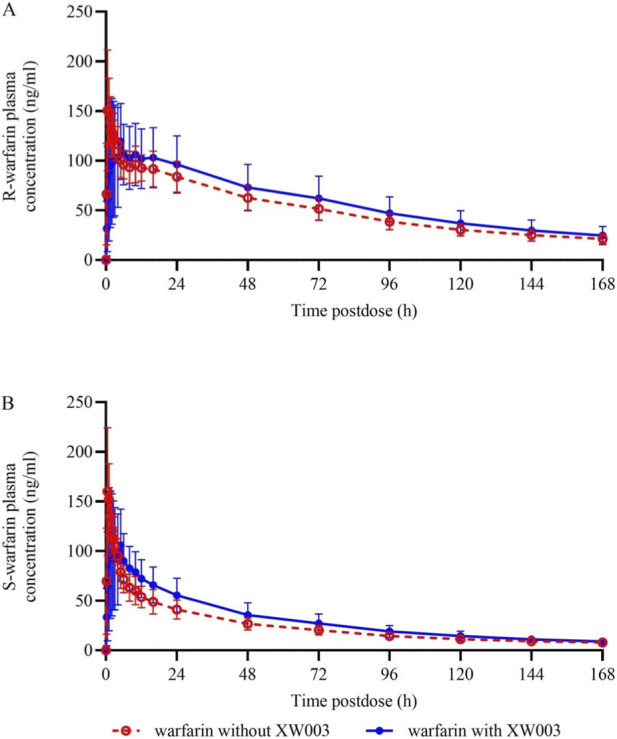
**(A, B)** Arithmetic mean plasma concentration–time profiles of **(A)** R-warfarin and **(B)** S-warfarin after a single dose of warfarin with or without XW003. Error bars represent standard deviation (SD).

For warfarin, blood samples were collected up to 168 h after a single oral dose, with or without XW003. Plasma S- and R-warfarin concentrations were measured by LC-MS/MS (LLOQ: 2.00 ng/mL for both enantiomers). As expected after a single dose, predose concentrations were below the LLOQ in both groups. Coadministration of XW003 markedly delayed the median T_max_ of S-warfarin from 0.50 h to 2.50 h and that of R-warfarin from 0.50 h to 3.21 h. By 168 h post-dose, plasma concentrations of both enantiomers were comparable between groups (Figure 3).

#### 3.2.3 XW003 PK

Blood samples for XW003 assay were collected from pre-dose up to 840 h after subcutaneous injection. XW003 concentrations in plasma were determined using LC-MS/MS (LLOQ: 2.00 ng/mL). Pre-dose levels were below the LLOQ, and steady state was achieved by the fourth dose. By 840 h after the last dose, concentrations had declined to near the LLOQ. The median Tmax,ss for XW003 was 23.94 h (range 23.88–96.48 h).

### 3.3 PK parameters analysis

All PK parameter analyses were conducted using the Pharmacokinetic Parameter Set (PKPS), defined as participants who received at least one dose of the investigational medical product(IMP) and had evaluable PK parameters. Six participants who experienced emesis within twice the median T_max_ following the last metformin dose during the XW003 coadministration phase had their corresponding PK parameters excluded from the primary analysis. Summaries of the PK parameters for metformin, warfarin, and steady-state XW003 are presented in Tables 3–5, respectively.

**TABLE 3:** Stable-state metformin plasma PK parameters with and without the coadministration of 1.2 mg XW003.

PK parameter	Metformin hydrochloride (N = 28)		Metformin hydrochloride + XW003 (N = 22)*		Values
	GM	CV%	GM	CV%	GMR % (90% CI)
C_max,ss_ (ng/mL)	1244.73	21.16	1004.93	37.34	80.64 (68.81, 94.49)
AUC_0-last,ss_ (h*ng/mL)	8588.23	15.38	8704.68	23.11	101.36 (91.70, 112.03)
AUC_0-inf,ss_ (h*ng/mL)	8996.90	16.87	9225.94	25.28	102.65 (92.53, 113.88)
t_1/2,ss_ (h)	8.06	40.25	7.02	35.87	​
C_min,ss_ (ng/mL)	154.75	37.75	231.24	49.44	​
C_avg,ss_ (ng/mL)	612.47	13.80	563.92	22.40	​
CL/F, ss (mL/h)	68030.62	13.82	73887.15	24.98	​
Vz/F, ss (mL)	791313.50	42.23	748448.31	47.13	​

GM: geometric mean; The GMR, is calculated as (metformin hydrochloride + XW003)/ (metformin hydrochloride alone); CV%: coefficient of variation percentage, CV% = (SD/Mean) * 100%.

* Six participants were excluded from the metformin PK, analysis due to emesis within twice the median T_max_ after the last dose; therefore, N = 22 for the metformin + XW003 group.

**TABLE 4:** S-warfarin and R-warfarin plasma PK parameters with and without the coadministration of 1.2 mg XW003.

PK parameter	Warfarin (N = 28)		Warfarin + XW003 (N = 28)		Values
	S-warfarin	R-Warfarin	S-warfarin	R-Warfarin	GMR% (90% CI)
	GM (CV%)	GM (CV%)	GM (CV%)	GM (CV%)	
C_max_ (ng/mL)	174.26 (27.00)	167.69 (24.97)	121.34 (40.64)	128.45 (35.77)	S-warfarin: 69.64 (56.89, 85.23) R-warfarin:76.60 (63.50, 92.39)
AUC_0-last_ (h*ng/mL)	4045.81 (19.86)	8470.62 (17.94)	4110.00 (35.40)	8883.57 (31.55)	S-warfarin: 101.59 (74.81, 137.94) R-warfarin:104.88 (87.94, 125.07)
AUC_0-inf_ (h*ng/mL)	4829.77 (18.82)	10893.49 (19.48)	4784.78 (34.20)	11324.44 (33.12)	S-warfarin: 99.07 (73.40, 133.71) R-warfarin: 103.96 (87.71, 123.21)
%AUCextrap	15.19 (33.15)	20.71 (34.53)	13.58 (31.44)	20.94 (21.38)	​
t_1/2_, (h)	65.97 (27.46)	75.71 (34.27)	53.61 (29.76)	73.67 (13.65)	​
CL/F (mL/h)	517.62 (21.16)	229.49 (19.52)	522.49 (456.76)	220.76 (197.53)	​
Vz/F (mL)	49267.55 (31.11)	25066.68 (27.34)	40413.95 (83.28)	23462.25 (184.94)	​
λz (1/h)	0.01 (23.87)	0.01 (23.50)	0.01 (264.52)	0.01 (14.38)	​

GM, geometric mean; The GMR, is calculated as (Warfarin + XW003)/ (Warfarin alone). CV%: coefficient of variation percentage, CV% = (SD/Mean) * 100%.

**TABLE 5:** Stable-state PK parameters of 1.2 mg XW003.

PK parameter	XW003 (N = 28)	
	GM	CV%
C_max_,ss (ng/mL)	228.88	16.80
AUC_0-last,ss_ (h*ng/mL)	82607.01	15.09
AUC_0-inf,ss_ (h*ng/mL)	83560.78	15.20
t_1/2,ss_ (h)	135.61	7.24
C_min,ss_ (ng/mL)	135.17	15.24
C_avg,ss_ (ng/mL)	190.24	14.61
CL/F,ss (mL/h)	37.55	14.96
Vz/F,ss (mL)	7345.50	14.27
λz_,ss_ (1/h)	0.01	7.28
DF (%)	47.98	22.60

GM, geometric mean; CV%: coefficient of variation percentage, CV% = (SD/Mean) * 100%.

The primary PK parameter for metformin, AUC_0–inf,ss_, was analyzed using a mixed-effects model (see Table 3). Comparison between metformin co-administered with XW003 (N = 22) and metformin alone (N = 28) showed a GMR for AUC_0–inf,ss_ of 102.65% (90% CI: 92.53%, 113.88%), which was entirely within the pre-specified no-effect interval (80%–125%). For the secondary PK parameters, the GMR for C_max,ss_ was 80.64% (90% CI: 68.81%, 94.49%), representing a 19% reduction, and the GMR for AUC_0–last,ss_ was 101.36% (90% CI: 91.70%, 112.03%), also within the no-effect interval. The median Tmax,ss for metformin was 1.48 h (range 0.73–3.00 h) when administered alone and 1.74 h (range 0.48–5.98 h) when coadministered with XW003, representing a delay of approximately 0.26 h. Although XW003 delayed metformin absorption (lower early concentrations, delayed T_max_), the overall exposure (AUC) remained unchanged, indicating that the extent of absorption was unaffected and the delay is unlikely to be clinically meaningful.

The primary PK parameters (AUC_0–inf_) for S- and R-warfarin were analyzed using a mixed-effects model (see Table 4). For S-warfarin, the GMR (warfarin + XW003/warfarin) was 99.07% (90% CI: 73.40%, 133.71%). Although the point estimate was within the 80%–125% no-effect interval, the 90% CI exceeded the predefined no-effect boundaries. However, the clinically more relevant PD endpoints (INR_max_ and AUC_INR_), which directly reflect warfarin’s anticoagulant effect, remained entirely within the no-effect bounds when coadministered with XW003 (see Section 3.4; Supplementary Table S5 for details). For R-warfarin, the GMR was 103.96% (90% CI: 87.71%, 123.21%), which was entirely within the no-effect interval.

For the secondary PK parameters (see Table 4), the GMR for S-warfarin C_max_ was 69.64% (90% CI: 56.89%, 85.23%), representing a 30% reduction. The GMR for S-warfarin AUC_0–last_ was 101.59% (90% CI: 74.81%, 137.94%); the point estimate was within the no-effect range, but the 90% CI extended beyond it. The GMR for R-warfarin AUC_0–last_ was 104.88% (90% CI: 87.94%, 125.07%). The lower limit of the confidence interval was within the no-effect range, while the upper limit marginally exceeded 125%.

### 3.4 PD analysis

**FIGURE 4:**
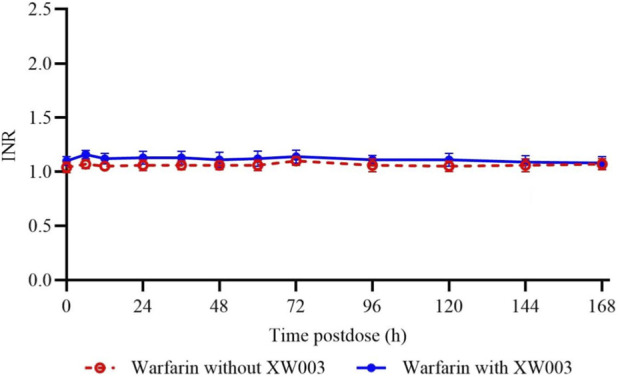
Mean INR profile for warfarin. Error bars represent standard deviation (SD).

To comprehensively evaluate the clinical impact of XW003 coadministration on warfarin’s anticoagulant effect, a PD analysis was performed. Based on PDPS, blood samples were collected within 168 h after a single dose of warfarin. Under both conditions (with and without XW003 coadministration), INR values remained within the normal reference range (0.8–1.5), with minimal fluctuation observed throughout the sampling period. A summary of warfarin PD parameters is provided in Supplementary Table S2. Mean INR profile for warfarin is provided in Figure 4.

The PD parameters of warfarin (INR_max_ and AUCINR,0-last) were analyzed using a mixed-effects model. After coadministration of warfarin with XW003 compared to warfarin alone, the GMR and 90% CI for INR_max_ were 105.63% (104.32%, 106.97%), and for AUCINR,0-last was 104.64% (103.74%, 105.54%). Both GMRs and their corresponding 90% CIs lay entirely within the pre-specified no-effect interval of 80%–125%.

### 3.5 Safety and tolerability

No deaths or serious adverse events (AEs) were reported, and all participants completed the treatment and the study. A total of 266 AEs were reported in all 28 participants (100%), all of which were mild or moderate in severity. The incidence of AEs varied by treatment phase: 16 participants (57.14%) experienced 29 AEs during metformin monotherapy; 9 (32.14%) reported 10 AEs during warfarin monotherapy; 26 (92.86%) had 156 AEs during XW003 monotherapy; 18 (64.29%) experienced 48 AEs during the XW003 and metformin combination phase, including 29 gastrointestinal AEs; and 13 participants (46.43%) reported 23 AEs during the XW003 and warfarin combination phase. Most AEs throughout the study were Grade 1. One participant (3.57%) experienced a single Grade 3 AE (syncope). By the end of the trial, the majority of AEs (93.23%; 248/266) had resolved, with the remainder (e.g., weight loss) not resolved at study completion. For clarity and conciseness, Table 6 summarizes AEs by System Organ Class (SOC) and Preferred Term (PT) that were reported at an incidence of ≥10% in any treatment group, thus focusing on the most frequently observed events.

**TABLE 6:** Treatment-emergent adverse events (occurring at a frequency of ≥10% in any treatment group) by system organ class.

SOC/P T	Metformin (N = 28)	Warfarin (N = 28)	XW003 (N = 28)	Metformin + XW003 (N = 28)	Warfarin + XW003 (N = 28)
Any adverse event	26/14 (50.00)	2/2 (7.14)	153/26 (92.86)	44/16 (57.14)	12/9 (32.14)
Gastrointestinal disorders	10/7 (25.00)	1/1 (3.57)	105/20 (71.43)	29/15 (53.57)	2/2 (7.14)
Diarrhoea	6/6 (21.43)	0/0 (0.00)	20/10 (35.71)	12/11 (39.29)	2/2 (7.14)
Nausea	1/1 (3.57)	0/0 (0.00)	30/9 (32.14)	4/4 (14.29)	0/0 (0.00)
Vomiting	0/0 (0.00)	0/0 (0.00)	10/6 (21.43)	10/10 (35.71)	0/0 (0.00)
Abdominal distension	0/0 (0.00)	0/0 (0.00)	12/8 (28.57)	0/0 (0.00)	0/0 (0.00)
Hiccups	0/0 (0.00)	0/0 (0.00)	12/6 (21.43)	1/1 (3.57)	0/0 (0.00)
Constipation	0/0 (0.00)	0/0 (0.00)	4/4 (14.29)	0/0 (0.00)	0/0 (0.00)
Abdominal pain	0/0 (0.00)	0/0 (0.00)	3/3 (10.71)	2/2 (7.14)	0/0 (0.00)
Investigations	11/8 (28.57)	1/1 (3.57)	29/24 (85.71)	9/6 (21.43)	6/6 (21.43)
Weight loss	0/0 (0.00)	0/0 (0.00)	22/22 (78.57)	1/1 (3.57)	1/1 (3.57)
ALT increased	5/5 (17.86)	0/0 (0.00)	0/0 (0.00)	3/3 (10.71)	0/0 (0.00)
AST increased	2/2 (7.14)	0/0 (0.00)	0/0 (0.00)	3/3 (10.71)	0/0 (0.00)
Blood uric acid increased	3/3 (10.71)	0/0 (0.00)	1/1 (3.57)	0/0 (0.00)	0/0 (0.00)
Metabolism and nutrition disorders	5/3 (10.71)	0/0 (0.00)	15/7 (25.00)	0/0 (0.00)	0/0 (0.00)
Decreased appetite	5/3 (10.71)	0/0 (0.00)	15/7 (25.00)	0/0 (0.00)	0/0 (0.00)

Data are presented as E/N (%), where E is the number of events, N is the number of subjects, and % is the incidence. Only preferred terms with an incidence of ≥10% in any treatment group are displayed.

All participants provided blood samples for ADA analysis within 1 h before the first dose and at 840 h (±24 h) after the final XW003 injection; all samples tested negative for ADA.

## 4 Discussion

This study presents a comprehensive clinical pharmacology evaluation of the novel GLP-1 receptor agonist XW003, with a specific focus on its drug-drug interaction (DDI) potential with two widely prescribed medications: metformin and the narrow therapeutic index drug warfarin. The study employed a fixed-sequence, crossover design that concurrently assessed the PK of both drugs and the PD of warfarin, providing clinically relevant data to support therapeutic decision-making. The key findings indicate that steady-state XW003 did not result in clinically significant PK or PD interactions with either metformin or warfarin.

For metformin, coadministration with XW003 did not significantly alter its total systemic exposure (AUC), as evidenced by the GMR and 90% confidence interval for AUC_0–inf,ss_ falling entirely within the 80%–125% no-effect interval. Although a modest decrease in geometric mean C_max,ss_ and a slight delay in median T_max_ were observed, a sensitivity analysis confirmed that vomiting did not substantially affect metformin AUC. These findings support the clinical relevance of XW003 in patients with type 2 diabetes, who are often prescribed metformin concomitantly. The maintained AUC supports the expectation that the efficacy of metformin, which is largely dependent on systemic exposure, would be preserved. Given that the reduction in C_max_ remained within the therapeutic window and gastrointestinal tolerability generally improves over time, the interaction is not considered clinically relevant, and no dose adjustment for metformin is required when coadministered with XW003.

For warfarin, a narrow therapeutic index drug, this study provides a thorough evaluation that includes both enantiomers and the key PD endpoint (INR). Coadministration with XW003 did not produce a clinically meaningful effect on the total systemic exposure (AUC) of either S- or R-warfarin. Although the 90% CIs for the primary PK parameters of S-warfarin slightly exceeded the 80%–125% boundary—a phenomenon occasionally observed in DDI studies involving highly variable drugs—this result should be interpreted in the context of the complete dataset. Notably, evaluation of the primary PD parameter showed no significant differences in INR_max_ or AUCINR between warfarin administered alone or with XW003, and all GMRs and 90% CIs were well within the no-effect bounds. This confirms that the minor PK changes did not lead to a clinically relevant PD effect. These results are consistent with, and extend, previous reports on other GLP-1 receptor agonists (e.g., lixisenatide (Maideen, 2019), dulaglutide (de la Peña et al., 2017; Anjum et al., 2024), semaglutide (Anjum et al., 2024), exenatide (Anjum et al., 2024), liraglutide (Anjum et al., 2024)) by providing a comprehensive dataset for this novel analog. Moreover, the overall PK and PD evidence aligns with regulatory guidelines (e.g., FDA/EMA DDI guidance), supporting a conclusion of no clinically significant interaction and offering clear guidance for both regulatory assessment and individualized prescribing. Therefore, no warfarin dose adjustment is necessary during coadministration with XW003, a relevant finding for safe use in patients requiring anticoagulation.

**FIGURE 5:**
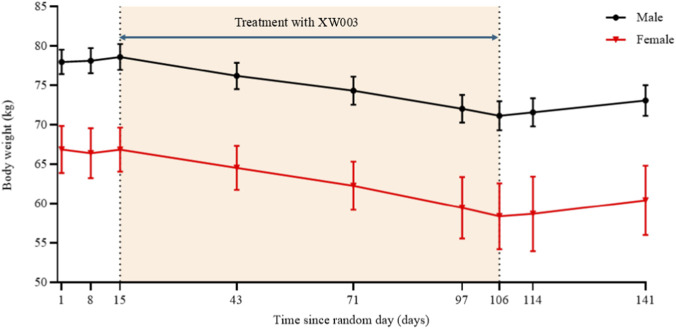
Mean change in body weight by sex during the treatment. Error bars represent standard deviation (SD).

We also considered whether the modest weight loss observed (approximately 4%–6% of total body weight, Figure 5) could have confounded the DDI assessment. However, if weight loss had compensated for a reduction in drug absorption, we would have expected to see a normal or even increased C_max_. Instead, we observed clear reductions in C_max_ (19% for metformin, 30% for S-warfarin) with delayed T_max_, which are characteristic of a mild delay in gastric emptying without clinically meaningful compensation. Therefore, the weight loss does not alter our conclusion of no clinically significant DDI.

We acknowledge a limitation of the present study design. As previously reported for other GLP-1 receptor agonists (e.g., dulaglutide and tirzepatide), delayed gastric emptying may be more pronounced after the first dose and could attenuate over time as tolerance develops. Our DDI evaluation was conducted at steady state (after 14 weeks of treatment). Therefore, we cannot rule out the possibility that the magnitude of interaction with concomitant oral medications might differ during the initial dose titration phase. Nevertheless, the observed changes in drug exposure (e.g., reduced C_max_ and delayed T_max_) were not considered clinically meaningful based on the PD assessment (INR for warfarin) and the wide therapeutic index of metformin. Notably, our steady-state findings are consistent with published data from steady-state semaglutide studies and single-dose dulaglutide studies, supporting the clinical applicability of our results.

Although our study did not directly evaluate oral contraceptives, the PK findings for metformin and warfarin–both orally administered drugs–suggest that XW003 has a mild effect on absorption rate (reduced C_max_, delayed T_max_) without altering total exposure (AUC). By analogy, the systemic exposure of oral contraceptive hormones, which determines contraceptive efficacy, is unlikely to be meaningfully reduced by XW003. Nonetheless, as a precaution, patients may consider additional non-hormonal contraceptive measures during the first few weeks of XW003 treatment until dedicated DDI studies are available.

We acknowledge a limitation regarding the timing of DDI assessment. As reported for other GLP?1 receptor agonists (e.g., dulaglutide and tirzepatide), delayed gastric emptying may be more pronounced after the first dose and could attenuate over time as tolerance develops. Our DDI evaluation was conducted at steady state (after 14 weeks of treatment); therefore, we cannot rule out the possibility that the magnitude of interaction with concomitant oral medications might differ during the initial dose titration phase. For narrow therapeutic index drugs such as warfarin, standard INR monitoring during the first 2–4 weeks of XW003 initiation is advisable, consistent with good clinical practice when introducing any new medication. For metformin, given its wide therapeutic index and the absence of a clinically meaningful interaction at steady state, no specific precaution is warranted.

The safety and tolerability profile of XW003 in this study was consistent with the known class effects of GLP-1 receptor agonists (Moiz et al., 2025) and further confirms its manageable safety profile under controlled clinical conditions. No serious adverse events, fatalities, or adverse events leading to permanent discontinuation were reported. The majority of adverse events were mild to moderate in severity. Gastrointestinal disorders (e.g., diarrhea, nausea, vomiting) and decreased appetite were the most commonly reported adverse events, and these were generally transient and self-limiting. No new safety signals were identified. As shown in Figure 5, body weight changes were analyzed separately by sex; no significant sex-based difference was observed in mean weight loss (two-tailed t-test, P > 0.05). The marked reduction in body weight following XW003 treatment—observed even in this non-obese healthy volunteer population over a limited duration—highlights its potent pharmacological activity and potential utility in weight management for indicated populations.

## 5 Conclusion

In conclusion, this drug-drug interaction study provides essential clinical pharmacology data for the novel GLP-1 analog XW003. It demonstrates that coadministration of XW003 has no clinically significant effect on the PK of metformin or warfarin or on the PD of warfarin. The safety profile of XW003 was consistent with that of the GLP-1 receptor agonist class. These robust findings support the concomitant use of XW003 with metformin or warfarin without dose adjustment in the intended patient populations, thereby supporting its safe use in real-world clinical practice where polypharmacy is common.

## Data Availability

The original contributions presented in the study are included in the article/Supplementary Material, further inquiries can be directed to the corresponding authors.

## References

[B1] Anjum P. Akbashev M. Utz A. Kisala S. (2024). Warfarin and GLP-1 receptor agonist interaction effects on time in therapeutic range. Blood 144 (Suppl. 1), 5579. 10.1182/blood-2024-211357

[B2] Bush M. Scott R. Watanalumlerd P. Zhi H. Lewis E. (2012). Effects of multiple doses of albiglutide on the PK, PD, and safety of digoxin, warfarin, or a low-dose oral contraceptive. Postgrad. Med. 124 (6), 55–72. 10.3810/pgm.2012.11.2613 23322139

[B3] Clark L. (2024). GLP-1 receptor agonists: a review of glycemic benefits and beyond. JAAPA 37 (4), 1–4. 10.1097/01.JAA.0001007388.97793.41 38531038

[B4] de la Peña A. Cui X. Geiser J. Loghin C. (2017). No dose adjustment is recommended for digoxin, warfarin, atorvastatin or a combination oral contraceptive when coadministered with dulaglutide. Clin. Pharmacokinet. 56 (11), 1415–1427. 10.1007/s40262-017-0531-7 28357715

[B5] Gabery S. Salinas C. G. Paulsen S. J. Ahnfelt-Rønne J. Alanentalo T. Baquero A. F. (2020). Semaglutide lowers body weight in rodents via distributed neural pathways. JCI Insight 5 (6), e133429. 10.1172/jci.insight.133429 32213703 PMC7213778

[B6] Guo W. Xu Z. Zou H. Li F. Li Y. Feng J. (2023). Discovery of ecnoglutide - a novel, long-acting, cAMP-biased glucagon-like peptide-1 (GLP-1) analog. Mol. Metab. 75, 101762. 10.1016/j.molmet.2023.101762 37364710 PMC10339203

[B7] He Y. Mi N. Cheng Z. Xue H. Han J. Wang H. (2025). Efficacy and safety of cAMP-biased GLP-1 receptor agonist ecnoglutide versus dulaglutide in patients with type 2 diabetes and elevated glucose concentrations on metformin monotherapy (EECOH-2): a 52-week, multicentre, open-label, non-inferiority, randomised, phase 3 trial. Lancet Diabetes Endocrinol. 13 (10), 863–873. 10.1016/S2213-8587(25)00196-2 40854315

[B8] Ji L. Gao L. Xue H. Tian J. Wang K. Jiang H. (2025). Efficacy and safety of a biased GLP-1 receptor agonist ecnoglutide in adults with overweight or obesity: a multicentre, randomised, double-blind, placebo-controlled, phase 3 trial. Lancet Diabetes Endocrinol. 13 (9), 777–789. 10.1016/S2213-8587(25)00141-X 40555243

[B9] Kimmel S. E. (2008). Warfarin therapy: in need of improvement after all these years. Expert Opin. Pharmacother. 9 (5), 677–686. 10.1517/14656566.9.5.677 18345947 PMC2855533

[B10] Maideen N. (2019). Pharmacologically relevant drug interactions of glucagon-like peptide-1 receptor agonists. J. Anal. Pharm. Res. 8 (2), 51–53. 10.15406/japlr.2019.08.00311

[B11] Moiz A. Filion K. B. Toutounchi H. Tsoukas M. A. Yu O. H. Y. Peters T. M. (2025). Efficacy and safety of glucagon-like peptide-1 receptor agonists for weight loss among adults without diabetes: a systematic review of randomized controlled trials. Ann. Intern Med. 178 (2), 199–217. 10.7326/annals-24-01590 39761578

[B12] Monney M. Jornayvaz F. R. Gariani K. (2023). GLP-1 receptor agonists effect on cognitive function in patients with and without type 2 diabetes. Diabetes Metab. 49 (5), 101470. 10.1016/j.diabet.2023.101470 37657738

[B13] Müller T. D. Finan B. Bloom S. R. D'Alessio D. Drucker D. J. Flatt P. R. (2019). Glucagon-like peptide 1 (GLP-1). Mol. Metab. 30, 72–130. 10.1016/j.molmet.2019.09.010 31767182 PMC6812410

[B14] Najafi S. Bahrami M. Butler A. E. Sahebkar A. (2022). The effect of glucagon-like peptide-1 receptor agonists on serum uric acid concentration: a systematic review and meta-analysis. Br. J. Clin. Pharmacol. 88 (8), 3627–3637. 10.1111/bcp.15344 35384008

[B15] Urquhart S. Willis S. (2020). Long-acting GLP-1 receptor agonists: findings and implications of cardiovascular outcomes trials. JAAPA 33 (S8 Suppl. 1), 19–30. 10.1097/01.JAA.0000669452.63883.45 32740122

[B16] Yao H. Zhang A. Li D. Wu Y. Wang C. Z. Wan J. Y. (2024). Comparative effectiveness of GLP-1 receptor agonists on glycaemic control, body weight, and lipid profile for type 2 diabetes: systematic review and network meta-analysis. BMJ 384, e076410. 10.1136/bmj-2023-076410 38286487 PMC10823535

[B17] Zhu D. Wang W. Tong G. Ma G. Ma J. Han J. (2024). Efficacy and safety of GLP-1 analog ecnoglutide in adults with type 2 diabetes: a randomized, double-blind, placebo-controlled phase 2 trial. Nat. Commun. 15 (1), 8408. 10.1038/s41467-024-52353-y 39333121 PMC11437099

