# Supplementary material for: No dose adjustment required for warfarin or metformin when coadministered with the novel GLP-1 receptor agonist ecnoglutide: an open-label, fixed-sequence, crossover study
Source: Front Pharmacol. 2026 Apr 29;17:1816593. doi: 10.3389/fphar.2026.1816593 (PMC13168755; doi:10.3389/fphar.2026.1816593)
Supplement: Supplementary file 1 [file Supplementaryfile1.docx]

## Method for XW003:

## Instrument

| Chromatography System | HPLC Pump: Shimadzu LC-30AD, Shimadzu LC-30AD |
| --- | --- |
|  | - Controller: Shimadzu CBM-20A lite |
|  | Degasser: Shimadzu DGV-20A5R |
|  | Column Oven: Shimadzu CTO-20A |
| Autosampler | Shimadzu SIL-30ACMP |
| Mass Spectrometer | TRIPLE QUAD 6500^+^, Applied Biosystems/ MDS Sciex |
| Ion Source | Electrospray Ionization (ESI) |
| Data Acquisition and Processing Software | Analyst 1.7.2 or equivalent, Applied Biosystems, U.S.A |
|  | Watson LIMS Version 7.6.1 or equivalent  Thermo Fisher Corporation, U.S.A |
|  | Microsoft Office 2013 or equivalent, Microsoft, U.S.A |

## Sample treatment

| 1 | Samples pretreatment (in 2.2 mL 96-well polypropylene plates, at room temperature, under yellow light):  a) For calibration standards, quality control samples, equilibration samples (excluding equilibration blank), system suitability samples, and test samples, add the corresponding sample (100 µL) and internal standard working solution (50.0 µL, 237 ng/mL).  b) For double blank samples and equilibration blank samples, add blank matrix (100 µL) and 50% ACN with 0.1% FA (50.0 µL).  c) For blank quality control samples (QC0), add blank matrix (100 µL) and internal standard working solution (50.0 µL, 237 ng/mL).  d) For ULOQ without IS samples, add the spiked ULOQ sample (100 µL) and 50% ACN with 0.1% FA (50.0 µL). |
| --- | --- |
| 2 | After vortexing the above sample plate for at least 1 minute, add 350 µL of MeOH to all samples. Vortex for at least 5 minutes to ensure thorough mixing. |
| 3 | Centrifuge the sample plate at 4000 rpm for 5 minutes at 4°C. |
| 4 | Transfer 250 µL of the supernatant to another clean 96-well polypropylene plate. Add 250 µL of 20% ammonium hydroxide to all samples, then vortex for at least 1 minute to ensure thorough mixing. |
| 5 | Condition the 96-well SPE plate (Oasis^®^ MAX, 96-well Solid Phase Extraction Plate, 30 µm, 10 mg, Waters):  Add 600 µL of MeOH to the 96-well SPE plate.  Add 600 µL of 10% ammonium hydroxide to the 96-well SPE plate. |
| 6 | Transfer all the mixed samples from Step 4 to the conditioned 96-well SPE plate from Step 5. Apply positive pressure if necessary to ensure complete passage through the plate. |
| 7 | Wash the 96-well SPE sample plate once with 600 µL of 10% ammonium hydroxide. Apply positive pressure if necessary. |
| 8 | Wash the 96-well SPE sample plate twice with 600 µL of ACN: IPA: water (75:5:20, v/v/v). Apply positive pressure if necessary. |
| 9 | Elute twice with 75.0 µL of ACN: IPA: water: FA (70:25:5:5, v/v/v/v). Collect the eluate. Apply positive pressure if necessary. |
| 10 | Add 600 µL of 40% ACN to all samples. Vortex for at least 5 minutes, then transfer to the autosampler. |

**Note: Unless otherwise specified, all sample processing steps above are performed at room temperature and under yellow light.**

# Analytical Condition

## HPLC Condition

| Column | ACQUITY UPLC^®^ Peptide BEH C18 Column, 2.1*50mm, 300Å, 1.7μm, Waters | | | | |
| --- | --- | --- | --- | --- | --- |
| Column Temperature | 50°C | | | | |
| Autosampler Temperature | 8℃ | | | | |
| Mobile Phase | Mobile Phase A: 100% Water with 0.5% FA  Mobile Phase B: 100% ACN with 0.5% FA | | | | |
| Mobile Phase Flow Rate  Initial gradient | 0.300 mL/min  35% Mobile Phase B | | | | |
| Gradient Program | Time (min) | Mode | | Event | Parameter |
|  | 5.00 | Pumps | | Pump B Conc. | 45 |
|  | 5.10 | Pumps | | Pump B Conc. | 80 |
|  | 5.70 | Pumps | | Pump B Conc. | 80 |
|  | 5.80 | Pumps | | Pump B Conc. | 10 |
|  | 6.10 | Pumps | | Pump B Conc. | 10 |
|  | 6.20 | Pumps | | Pump B Conc. | 80 |
|  | 6.21 | Autosampler | | Rinse | |
|  | 6.80 | Pumps | | Pump B Conc. | 80 |
|  | 6.90 | Pumps | | Pump B Conc. | 10 |
|  | 7.20 | Pumps | | Pump B Conc. | 10 |
|  | 7.30 | Pumps | | Pump B Conc. | 80 |
|  | 8.00 | Pumps | | Pump B Conc. | 80 |
|  | 8.10 | Pumps | | Pump B Conc. | 35 |
|  | 8.80 | System Controller | | Stop |  |
| Diverter valve | Time (min) | | Note | | |
|  | 0.00~2.80 | | Flow to waste liquid | | |
|  | 2.80~5.30 | | Flow to mass spectrometer | | |
|  | 5.30~8.80 | | Flow to waste liquid | | |
| Autosampler needle wash | Needle Wash Solution (R0, R1, R2): ACN: MeOH: IPA: Water: TFA (25:25:25:25:0.1, v/v/v/v/v)  Needle Wash Solution (R3): IPA: Water: TFA (50:50:0.1, v/v/v)  Sampling speed: 5.0 µL/sec  Measuring Line Purge Volume: 100 µL  Air Gap: off  Rinse Type: External  Rinsing Speed: 35 µL/sec  Rinse Port liquid Selection: R1  Rinsing Volume: 1000 µL  Rinse Mode: Before and after aspiration  Rinse Time: 2 sec  Rinse Method: Rinse Pump Then port)  Rinse Dip Time: 2 sec | | | | |
| Injection Volume | 2.00 µL | | | | |
| Retention Time | XW003: at approximately 3.65 min M0: at approximately 4.49 min | | | | |

**Note: HPLC conditions could be modified if necessary.**

## MS Condition

| Ionization mode | ESI, positive, MRM | | |
| --- | --- | --- | --- |
| Source Parameters | CUR: 35 psi | | |
|  | GS1: 50 psi | | |
|  | GS2: 70 psi | | |
|  | IonSpray Voltage: 5500 V | | |
|  | TEM: 400°C | | |
|  | Resolution Q1/Q3: Unit/ Low | | |
|  | CAD: 10 unit | | |
|  | MR Pause: 20 msec | | |
| Acquisition Time | 8.80 min | | |
| Selective Reaction Monitor |  | XW003 | M0 |
|  | m/z | 857.900 / 1071.400 | 1050.800 /1338.800 |
|  | DP | 60.00V | 100.00V |
|  | CE | 38.00eV | 46.00eV |
|  | EP | 10.00V | 6.00V |
|  | CXP | 30.00V | 30.00V |
|  | Dwell | 150 msec | 150 msec |

**Note: The MS conditions above may be modified if necessary.**

# Calibration Regression

| Regression Mode | y = ax + b, linear regression |
| --- | --- |
| Weighing Factor | 1/x^2^ |
| y | Peak area ratio of analyte to internal standard |
| x | Concentration of analyte in C standards |

## Method for Metformin:

## Instrument

| Chromatography System | HPLC Pumps: Shimadzu LC-20AD, Shimadzu LC-20AD  Controller: Shimadzu CBM-20A lite or Shimadzu CBM-20A  Degasser: Shimadzu DGU-20A_5_ or Shimadzu DGU-20A_5R_  Column Oven: Shimadzu CTO-20A |
| --- | --- |
| Autosampler | SIL-20AC _XR_ or SIL-20AC _HT_ |
| Mass Spectrometer | 4000 Q TRAP, Applied Biosystems / MDS Sciex |
| Ion Source | Electrospray Ionization (ESI) |
| Data Acquisition and Processing Software | Analyst 1.7.2 or equivalent, Applied Biosystems, U.S.A |
|  | Watson LIMS Version 7.6.1 or equivalent  Thermo Fisher Corporation, U.S.A |
|  | Microsoft Office 2013 or equivalent, Microsoft, U.S.A |

## Sample treatment

| 1 | Samples pretreatment (in 2.2 mL 96-well polypropylene plates, at room temperature, under yellow light):  a) For calibration standards, quality control samples, and test samples, add the corresponding sample (50.0 µL) and internal standard working solution (50.0 µL, 200 ng/mL).  b) For double blank samples, add blank matrix (50.0 µL) and 50% MeOH (50.0 µL).  c) For blank quality control samples (QC0), add blank matrix (50.0 µL) and internal standard working solution (50.0 µL, 200 ng/mL).  d) For ULOQ without IS samples, add the mixed ULOQ sample (50.0 µL) and 50% MeOH (50.0 µL). |
| --- | --- |
| 2 | After vortexing the above sample plate for at least 1 minute, add 400 µL of ACN to all samples. Vortex to mix thoroughly for at least 10 minutes. |
| 3 | Centrifuge the sample plate at 4000 rpm for 10 minutes at 4°C. |
| 4 | Transfer 50.0 µL of the supernatant to another clean 96-well polypropylene plate. Add 450 µL of 90% ACN containing 10 mM NH4Ac and 1% FA to all samples. Vortex to mix thoroughly for at least 10 minutes. |

**Note: Unless otherwise specified, all sample processing steps above are performed at room temperature and under yellow light.**

# Analytical Condition

## HPLC Condition

| Column | Atlantis^®^ HILIC Silica (5µm, 2.1×50mm) , Waters | | | | |
| --- | --- | --- | --- | --- | --- |
| Column Temperature | 40°C | | | | |
| Autosampler Temperature | 8℃ | | | | |
| Mobile Phase | Mobile Phase A: 100% Water with 10 mM NH₄Ac and 0.1% FA  Mobile Phase B: 90% ACN with 10 mM NH₄Ac and 1% FA | | | | |
| Mobile Phase Flow Rate  Initial gradient | 0.600 mL/min  100% Mobile Phase B | | | | |
| Gradient Program | Time (min) | Mode | | Event | Parameter |
|  | 1.20 | Pumps | | Pump B Conc. | 100 |
|  | 1.21 | Pumps | | Pump B Conc. | 50 |
|  | 2.20 | Pumps | | Pump B Conc. | 50 |
|  | 2.21 | Pumps | | Pump B Conc. | 100 |
|  | 4.00 | System Controller | | Stop |  |
| Diverter valve | Time (min) | | Note | | |
|  | 0.0~0.2 | | Flow to waste liquid | | |
|  | 0.2~2.0 | | Flow to mass spectrometer | | |
|  | 2.0~4.0 | | Flow to waste liquid | | |
| Autosampler needle wash | Needle Wash Solution 1: MeOH: ACN: IPA: Water: NH₄OH (10:10:10:5:5, v/v/v/v/v)  Needle Wash Solution 2: 100% MeOH  Sampling speed: 5.0 µL/sec  Rinsing Speed: 35 µL/sec  Rinsing Volume: 500 µL  Rinse Mode) : Before and after aspiration  Rinse Method: Rinse Pump Then Port  Rinse Time:2 sec  Rinse Dip Time: 2 sec | | | | |
| Injection Volume | 10.0 µL | | | | |
| Retention Time | Metformin: at approximately 1.20 min; Metformin-d6: at approximately 1.20 min | | | | |

**Note: HPLC conditions could be modified if necessary.**

## MS Condition

| Ionization mode | ESI, positive, MRM | | |
| --- | --- | --- | --- |
| Source Parameters | CUR: 30 psi | | |
|  | GS1: 50 psi | | |
|  | GS2: 50 psi | | |
|  | IonSpray Voltage: 3000 V | | |
|  | TEM: 500°C | | |
|  | Ihe: ON | | |
|  | Resolution Q1/Q3: Unit/ Unit | | |
|  | CAD: 6.00 unit | | |
|  | MR Pause: 20 msec | | |
| Acquisition Time | 4.0 min | | |
| Selective Reaction Monitor |  | Metformin | Metformin-d6 |
|  | m/z | 130.100/71.100 | 136.100/77.100 |
|  | DP | 40.00 V | 40.00 V |
|  | CE | 50.00 eV | 50.00 eV |
|  | EP | 10.00 V | 10.00 V |
|  | CXP | 14.00 V | 14.00 V |
|  | Dwell | 200 msec | 200 msec |

**Note: The MS conditions above may be modified if necessary.**

# Calibration Regression

| Regression Mode | y = ax + b, linear regression |
| --- | --- |
| Weighing Factor | 1/x^2^ |
| y | Peak area ratio of analyte to internal standard |
| x | Concentration of analyte in C standards |

## Method for R- Warfarin and S- Warfarin:

## Instrument

| Chromatography System | HPLC Pumps: Shimadzu LC-20AD, Shimadzu LC-20AD |
| --- | --- |
|  | Controller: Shimadzu CBM-20A lite |
|  | Degasser: Shimadzu DGV-20A5R |
|  | Column Oven: Shimadzu CTO-20A |
| Autosampler | Shimadzu SIL-20AC Autosampler |
| Mass Spectrometer | TRIPLE QUAD 4000, Applied Biosystems/ MDS Sciex |
| Ion Source | Electrospray Ionization (ESI) |
| Data Acquisition and Processing Software | Analyst 1.7.2 or equivalent, Applied Biosystems, U.S.A |
|  | Watson LIMS Version 7.6.1 or equivalent  Thermo Fisher Corporation, U.S.A |
|  | Microsoft Office 2013 or equivalent, Microsoft, U.S.A |

## Sample treatment

| 1 | Samples pretreatment (in 2.2 mL 96-well polypropylene plates):  a) For calibration standards, quality control samples, and test samples, mix the sample (50.0 µL) with internal standard working solution (50.0 µL, 600 ng/mL).  b) For double blank samples, mix blank matrix (50.0 µL) with 50% MeOH (50.0 µL).  c) For control blank samples (QC0), mix blank matrix (50.0 µL) with internal standard working solution (50.0 µL, 600 ng/mL).  d) For ULOQ without IS samples, mix the ULOQ sample (50.0 µL) with 50% MeOH (50.0 µL). |
| --- | --- |
| 2 | After vortexing the above sample plate for at least 1 minute, add 400 µL of ACN to all samples. Vortex to mix thoroughly for at least 10 minutes. |
| 3 | Centrifuge the sample plate at 4000 rpm for 10 minutes at 4°C. |
| 4 | Transfer 200 µL of the supernatant to another clean 96-well polypropylene plate. Add 200 µL of 100% Water to all samples. Vortex to mix thoroughly for at least 5 minutes, then transfer to the autosampler. |

**Note: Unless otherwise specified, all sample processing steps above are performed at room temperature and under yellow light.**

## Analytical Condition

## HPLC Condition

| Column | CHIRALCEL^®^ OJ-3R (3µm, 4.6×150 mm) , DAICEL CORPORATION (No guard column used) | | | | |
| --- | --- | --- | --- | --- | --- |
| Column Temperature | 40℃ | | | | |
| Autosampler Temperature | 8℃ | | | | |
| Mobile Phase | Mobile Phase A: 100% Water with 5 mM NH₄Ac and 0.1% AA  Mobile Phase B: 100% ACN | | | | |
| Mobile Phase Flow Rate | 0.800 mL/min | | | | |
| Initial gradient | 55% Mobile Phase B | | | | |
| Gradient Program | Time (min) | Mode | | Event | Parameter |
|  | 5.60 | Pumps | | Pump B Conc. | 55 |
|  | 5.70 | Pumps | | Pump B Conc. | 95 |
|  | 7.70 | Pumps | | Pump B Conc. | 95 |
|  | 7.80 | Pumps | | Pump B Conc. | 55 |
|  | 10.0 | System Controller | | Stop |  |
| Diverter valve | Time (min) | | Note | | |
|  | 0.0~3.5 | | Flow to waste liquid | | |
|  | 3.5~7.0 | | Flow to mass spectrometer | | |
|  | 7.0~10.0 | | Flow to waste liquid | | |
| Autosampler needle wash | Needle Wash Solution: 100% ACN  Sampling Speed: 5.0 µL/sec  Measuring Line Purge Volume: 100 µL  Air Gap: off  Rinsing Speed: 35 µL/sec  Rinse Port Liquid Selection: R1  Rinsing Volume: 300 µL  Rinse Mode: Before and after aspiration  Rinse Time: 2 sec  Rinse Method: Rinse Port Only  Rinse Dip Time: 2 sec | | | | |
| Injection Volume | 25.0 µL | | | | |
| Retention Time | R-Warfarin: at approximately 4.9 min; S-Warfarin: at approximately 5.6 min  R-Warfarin-d5: at approximately 4.9 min; S-Warfarin-d5: at approximately 5.6 min | | | | |

**Note: HPLC conditions could be modified if necessary.**

## MS Condition

| Ionization mode | ESI, positive, MRM | | |
| --- | --- | --- | --- |
| Source Parameters | CUR: 35 psi | | |
|  | GS1: 40 psi | | |
|  | GS2: 50 psi | | |
|  | IonSpray Voltage: 5000 V | | |
|  | TEM: 500°C | | |
|  | ihe: ON | | |
|  | Resolution Q1/Q3: Unit/ Unit | | |
|  | CAD: 8.00 unit | | |
|  | MR Pause: 20 msec | | |
| Acquisition Time | 10.0 min | | |
| Selective Reaction Monitor |  | Warfarin# | Warfarin-d5# |
|  | m/z | 309.300 / 163.000 | 314.200 /163.000 |
|  | DP | 50.00 V | 50.00 V |
|  | CE | 20.00 eV | 20.00 eV |
|  | EP | 10.00 V | 10.00 V |
|  | CXP | 15.00 V | 15.00 V |
|  | Dwell | 300 msec | 300 msec |

**Note: R-Warfarin and S-Warfarin, as well as R-Warfarin-d_5_ and S-Warfarin-d5_,_ share the same monitoring ion pairs and mass spectrometry parameters. The isomers are separated by liquid chromatography. When creating the acquisition method in Analyst software, common ion pairs are set for monitoring. For quantitation, the method is configured separately based on their respective peaks to complete the quantitative analysis. The mass spectrometry operating conditions above may be modified if necessary.**

# Calibration Regression

| Regression Mode | y = ax + b, linear regression |
| --- | --- |
| Weighing Factor | 1/x^2^ |
| y | Peak area ratio of analyte to internal standard |
| x | Concentration of analyte in C standards |
