# Supplementary material for: No dose adjustment required for warfarin or metformin when coadministered with the novel GLP-1 receptor agonist ecnoglutide: an open-label, fixed-sequence, crossover study
Source: Front Pharmacol. 2026 Apr 29;17:1816593. doi: 10.3389/fphar.2026.1816593 (PMC13168755; doi:10.3389/fphar.2026.1816593)
Supplement: Supplementary file 2 [file Supplementaryfile2.docx]

**Table S1 Pharmacokinetic Blood Sampling Schedule of Metformin**

| No. | Nominal time | Collection Time Window | Volume Collected | Day of Collection | Scheduled  Timeᵇ |
| --- | --- | --- | --- | --- | --- |
| 1 | 0ᵃ | Within -1 h | 3 mL | D4/D100 | 7:00~7:59 |
| 2 | 30 min | ±2 min | 3 mL | D4/D100 | 8:30 |
| 3 | 45 min | ±2 min | 3 mL | D4/D100 | 8:45 |
| 4 | 1 h | ±2 min | 3 mL | D4/D100 | 9:00 |
| 5 | 1.5 h | ±2 min | 3 mL | D4/D100 | 9:30 |
| 6 | 2 h | ±2 min | 3 mL | D4/D100 | 10:00 |
| 7 | 2.5 h | ±2 min | 3 mL | D4/D100 | 10:30 |
| 8 | 3 h | ±2 min | 3 mL | D4/D100 | 11:00 |
| 9 | 4 h | ±5 min | 3 mL | D4/D100 | 12:00 |
| 10 | 5 h | ±5 min | 3 mL | D4/D100 | 13:00 |
| 11 | 6 h | ±5 min | 3 mL | D4/D100 | 14:00 |
| 12 | 8 h | ±5 min | 3 mL | D4/D100 | 16:00 |
| 13 | 10 h | ±5 min | 3 mL | D4/D100 | 18:00 |
| 14 | 12 h | ±5 min | 3 mL | D4/D100 | 20:00 |
| 15 | 14 h | ±10 min | 3 mL | D4/D100 | 22:00 |
| 16 | 16 h | ±10 min | 3 mL | D5/D101 | 00:00 on Day 2 |
| 17 | 20 h | ±10 min | 3 mL | D5/D101 | 04:00 on Day 2 |
| 18 | 24 h | ±10 min | 3 mL | D5/D101 | 08:00 on Day 2 |
| 19 | 30 h | ±10 min | 3 mL | D5/D101 | 14:00 on Day 2 |

ᵃ: Before the administration of metformin on D4 and D100; all subsequent time points are post-administration of this dose.
ᵇ: Scheduled times are approximate times.

**Table S2 Pharmacokinetic and PD Blood Sampling Schedule of Warfarin**

| No. | Nominal time | Collection Time Window | Volume Collected | Day of Collection | Scheduled  Timeᵇ | PKᶜ | INRᶜ |
| --- | --- | --- | --- | --- | --- | --- | --- |
| 1 | 0ᵃ | Within -1 h | 3 mL + 2 mLᵈ | D8/D107 | 7:00~7:59 | PK | INR |
| 2 | 15 min | ±2 min | 3 mL | D8/D107 | 8:15 | PK | - |
| 3 | 30 min | ±2 min | 3 mL | D8/D107 | 8:30 | PK | - |
| 4 | 1 h | ±2 min | 3 mL | D8/D107 | 9:00 | PK | - |
| 5 | 1.5 h | ±2 min | 3 mL | D8/D107 | 9:30 | PK | - |
| 6 | 2 h | ±2 min | 3 mL | D8/D107 | 10:00 | PK | - |
| 7 | 2.5 h | ±2 min | 3 mL | D8/D107 | 10:30 | PK | - |
| 8 | 3 h | ±2 min | 3 mL | D8/D107 | 11:00 | PK | - |
| 9 | 4 h | ±5 min | 3 mL | D8/D107 | 12:00 | PK | - |
| 10 | 5 h | ±5 min | 3 mL | D8/D107 | 13:00 | PK | - |
| 11 | 6 h | ±5 min | 3 mL + 2 mLᵈ | D8/D107 | 14:00 | PK | INR |
| 12 | 8 h | ±5 min | 3 mL | D8/D107 | 16:00 | PK | - |
| 13 | 10 h | ±5 min | 3 mL | D8/D107 | 18:00 | PK | - |
| 14 | 12 h | ±5 min | 3 mL + 2 mLᵈ | D8/D107 | 20:00 | PK | INR |
| 15 | 16 h | ±10 min | 3 mL | D9/D108 | 00:00 on Day 2 | PK | - |
| 16 | 24 h | ±10 min | 3 mL + 2 mLᵈ | D9/D108 | 08:00 on Day 2 | PK | INR |
| 17 | 36 h | ±10 min | 2 mL | D9/D108 | 20:00 on Day 2 | - | INR |
| 18 | 48 h | ±10 min | 3 mL + 2 mLᵈ | D10/D109 | 08:00 on Day 3 | PK | INR |
| 19 | 60 h | ±10 min | 2 mL | D10/D109 | 20:00 on Day 3 | - | INR |
| 20 | 72 h | ±10 min | 3 mL + 2 mLᵈ | D11/D110 | 08:00 on Day 4 | PK | INR |
| 21 | 96 h | ±1 h | 3 mL + 2 mLᵈ | D12/D111 | 08:00 on Day 5 | PK | INR |
| 22 | 120 h | ±1 h | 3 mL + 2 mLᵈ | D13/D112 | 08:00 on Day 6 | PK | INR |
| 23 | 144 h | ±1 h | 3 mL + 2 mLᵈ | D14/D113 | 08:00 on Day 7 | PK | INR |
| 24 | 168 h | ±1 h | 3 mL + 2 mLᵈ | D15/D114 | 08:00 on Day 8 | PK | INR |

ᵃ: Before the morning administration of warfarin on D8 and D107; all subsequent time points are post-administration of this dose.
ᵇ: Scheduled times are approximate times.
ᶜ: Samples for INR (International Normalized Ratio, PD index) are only collected at the time points marked with INR, and samples for PK are only collected at the time points marked with PK. If the collection time for PK and INR overlaps, PK samples shall be collected first.
ᵈ: 3 mL for PK blood sample collection and 2 mL for INR blood sample collection; the same below.

**Table S3 Pharmacokinetic Blood Sampling Schedule of XW003**

| No. | Nominal time | Collection Time Window | Volume Collected | Day of Collection | Scheduled  Time^c^ |
| --- | --- | --- | --- | --- | --- |
| 1 | 0 (Before the 1st administration) | Within -1 h | 3 mL | D15 | 7:00~7:59 |
| 2 | 0 (Before the 5th administration) | Within -1 h | 3 mL | D43 | 7:00~7:59 |
| 3 | 0 (Before the 9th administration) | Within -1 h | 3 mL | D71 | 7:00~7:59 |
| 4 | 0 (Before the 12th administration) | Within -1 h | 3 mL | D92 | 7:00~7:59 |
| 5 | 0 (Before the 13th administration) | Within -1 h | 3 mL | D99 | 7:00~7:59 |
| 6 | 24 h (After the 13th administration)ᵃ | ±10 min | 3 mL | D100 | 08:00 on Day 2 |
| 7 | 48 h (After the 13th administration) | ±10 min | 3 mL | D101 | 08:00 on Day 3 |
| 8 | 0 (Before the 14th administration)ⁿᵇ | Within -1 h | 3 mL | D106 | 7:00~7:59 |
| 9 | 24 h | ±10 min | 3 mL | D107 | 08:00 on Day 2 |
| 10 | 48 h | ±10 min | 3 mL | D108 | 08:00 on Day 3 |
| 11 | 72 h | ±10 min | 3 mL | D109 | 08:00 on Day 4 |
| 12 | 96 h | ±1 h | 3 mL | D110 | 08:00 on Day 5 |
| 13 | 120 h | ±1 h | 3 mL | D111 | 08:00 on Day 6 |
| 14 | 144 h | ±1 h | 3 mL | D112 | 08:00 on Day 7 |
| 15 | 168 h | ±1 h | 3 mL | D113 | 08:00 on Day 8 |
| 16 | 840 h | ±24 h | 3 mL | D141 | 08:00 on Day 36 |

ᵃ: 24 h after the 13th administration of XW003, collected before the administration of metformin.
ᵇ: Before the last administration of XW003 on D106 (Trial 1); all subsequent time points are post-administration of this dose.

^c^:Scheduled times are approximate times.
